# Innovating shorter, all-oral, precise, individualized treatment regimen for rifampicin-resistant tuberculosis (INSPIRE TB): study protocol for a pragmatic randomised controlled trial

**DOI:** 10.1186/s12879-026-13017-y

**Published:** 2026-03-17

**Authors:** Yilin Zhang, Yang Li, Yan Ren, Ting Li, Yajiao Xing, Jiqin Wu, Pengfei Ren, Feng Sun, Wenhong Zhang

**Affiliations:** 1 Shanghai Key Laboratory of Infectious Diseases and Biosafety Emergency Response, Department of Infectious Diseases, Shanghai Medical College, National Medical Center for Infectious Diseases, Huashan Hospital, Fudan University, Shanghai, China; 2 Shanghai Sci-Tech Inno Centre for Infection & Immunity, Shanghai, China; 3 Department of Tuberculosis, The Sixth People’ s Hospital of Zhengzhou, Zhengzhou, Henan China; 4 Present Address: National Medical Center for Infectious Diseases, Huashan Hospital, 12 Middle Wulumuqi Road, Jing’an District, Shanghai, China

**Keywords:** Rifampicin-resistant tuberculosis, Oral shorter regimen, Randomised controlled trial, Rapid drug susceptibility testing, Bedaquiline

## Abstract

**Background:**

Rifampicin-resistant tuberculosis (RR-TB) poses a great threat to global health. Increasing evidence of novel regimens in trial settings has risen, but the combinations of oral shorter regimens have not been optimised. An evaluation of the efficacy and safety of short-term regimens utilizing various drug combinations is essential across different settings.

**Methods:**

INSPIRE TB is a pragmatic, multicentre, randomised, controlled, non-inferiority open-label trial to evaluate novel regimens in pulmonary RR-TB patients of 16–75 years old with or without fluoroquinolone resistance. Before randomisation, an individualized assessment is conducted, considering drug susceptibility, contradictions, tolerance. Eligible participants are randomised based on fluoroquinolone susceptibility results from the Xpert MTB/XDR assay. Participants susceptible to fluoroquinolones are randomised to receive either one of seven different nine-month oral regimens or a nine-month standard-of-care regimen as the control. The experimental arms are five-drug regimens, each consisting of five agents selected from the following: bedaquiline, linezolid, a fluoroquinolone, cycloserine, clofazimine and pyrazinamide. Fluoroquinolone-resistant participants are randomised to either a 9-month oral regimen (bedaquiline, cycloserine, clofazimine, linezolid and pyrazinamide), or a 20-month conventional regimen as control in a 1:1 ratio. The primary outcome is the proportion of participants with a favourable outcome (two negative culture for *Mycobacterium Tuberculosis*, the latest sample collected between month 21 and 23) at 21 months after randomisation in the modified intention-to-treat population, measured in fluoroquinolone-susceptible and fluoroquinolone-resistant participants respectively. A sample size of 832 fluoroquinolone-susceptible RR-TB patients and 234 fluoroquinolone-resistant RR-TB patients afford 80% power to establish non-inferiority with a non-inferiority margin of 10% at a one-sided α level of 2.5%. The type I error will be controlled with a fixed-sequence approach.

**Discussion:**

Identifying safer and effective short regimens remains an obstacle for drug-resistant tuberculosis and more treatment options are needed to benefit patients from different countries and settings to reduce disease burden. The INSPIRE TB study hopes to provide robust evidence on various options of safer and effective 9-month oral regimens for RR-TB in China.

**Trail registration:**

ClinicalTrial.gov, NCT05081401. Registered on October 18, 2021; the record was lastupdated for study protocol version 6.0, on 06 April 2025.

**Supplementary Information:**

The online version contains supplementary material available at 10.1186/s12879-026-13017-y.

## Background

Rifampicin-resistant tuberculosis (RR-TB) is a growing public health threat, caused by *Mycobacterium Tuberculosis* (MTB) strains resistant to key first-line drugs. Globally, an estimate of 390,000 incident cases of multidrug-resistant or rifampicin-resistant tuberculosis (MDR/RR-TB) were reported in 2024, accounting for 3.2% of all TB cases [1–4]. Treatment of MDR/RR-TB requires regimens combining second-line or novel drugs. Although the global treatment success rate has gradually improved since 2012, it remained relatively low at 71% in 2022 [3].

Facing ongoing challenges in RR-TB treatment, the pursuit of shorter, safer, and more effective regimens remains a key research priority. In 2019, the landmark STREAM trial—the first randomised controlled trial evaluating shorter regimens for MDR-TB—demonstrated non-inferior efficacy of a standardised 9-month, seven-drug regimen compared to the longer regimen [5]. The subsequent Nix-TB trial reported a 90% treatment success rate using a 26-week regimen of bedaquiline, pretomanid and linezolid in patients with pre-extensively drug-resistant tuberculosis (Pre-XDR TB) [6]. Recent results from the STREAM stage 2 trial showed treatment success rates of 83% and 91% for a 9-month and a 6-month oral bedaquiline-containing regimens, respectively [7]. Additionally, the endTB trial tested four different 9-month oral regimens containing bedaquiline or delamanid, with success rates exceeding 80% [8]. Despite growing evidence supporting various RR-TB treatment options, it remains crucial to evaluate shorter oral regimens with various drug combinations across different settings.

Overall, the INSPIRE TB study represents the first multicentre, multi-arm randomised controlled trial in China designed to systematically evaluate the efficacy and safety of multiple oral shorter regimens for RR-TB, stratified by fluoroquinolone resistance. For fluoroquinolone-susceptible patients, seven 9-month oral regimens will be compared against a 9-month standardized regimen, while for fluoroquinolone-resistant patients, a 9-month bedaquiline-containing oral regimen will be compare with a conventional long regimen. Each experimental regimen consists of five drugs selected from the following agents: bedaquiline, linezolid, fluoroquinolones (moxifloxacin or levofloxacin), cycloserine, clofazimine and pyrazinamide. Additionally, the study will investigate a structured linezolid dose-adjustment strategy to determine its optimal dosing. It is expected that the INSPIRE TB trial will broaden the range of shorter, oral treatment options for patients with RR-TB.

## Methods

### Design

**Fig. 1:**
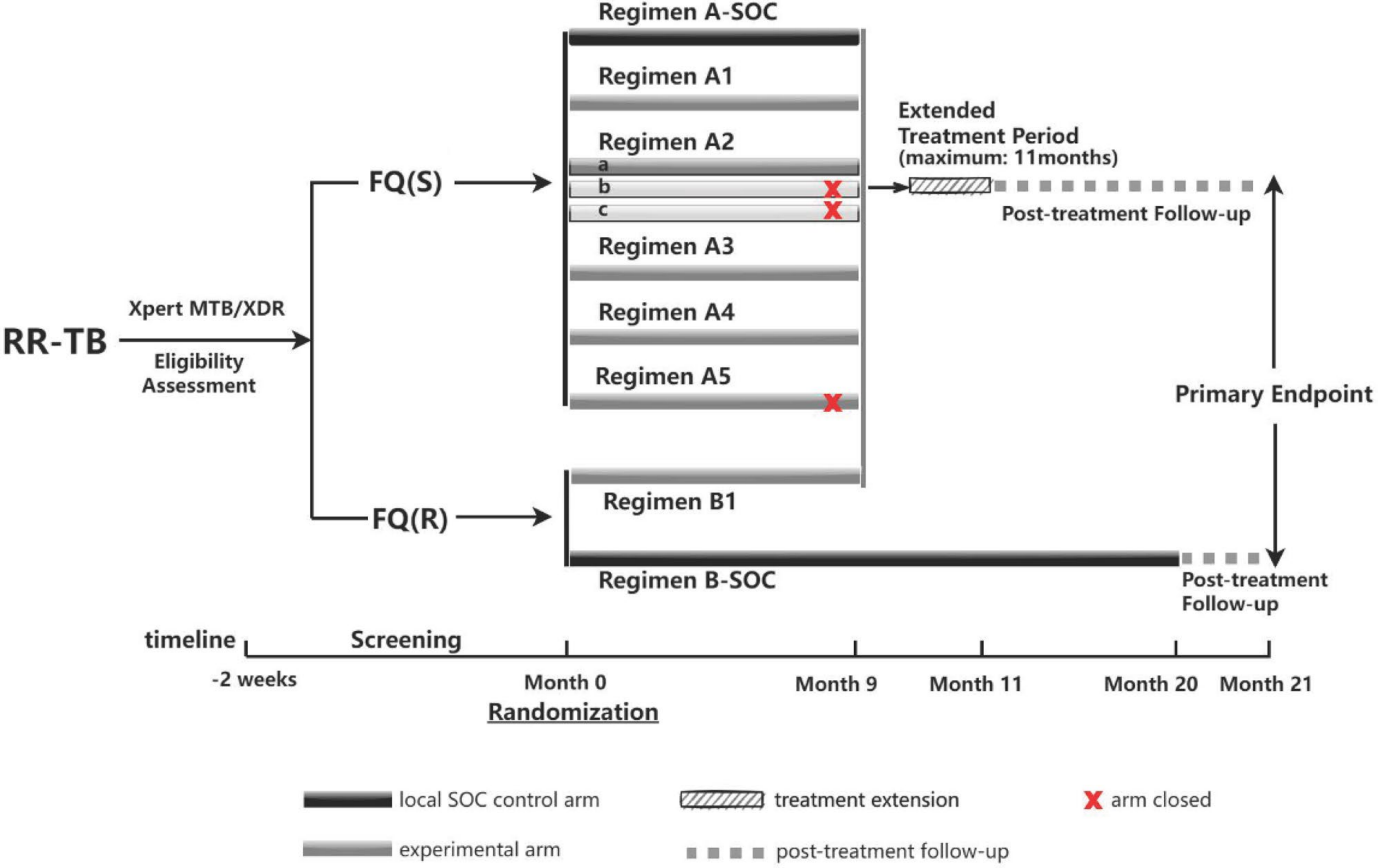
Study schematic. The INSPIRE TB study is a multicentre, open-label, randomised, pragmatic, non-inferiority trial recruiting rifampicin-resistant pulmonary tuberculosis patients aged 16–75 years old. Eligible participants will be divided into group **A** (fluoroquinolone susceptible) and group **B** (fluoroquinolone-resistant) according to the Xpert MTB/XDR assay. Recruitments of three experimental arms (A2b, A2c, A5) were ceased in protocol version 6.0 (Nov 2024). Abbreviations: RR-TB, rifampicin-resistant tuberculosis; FQ(S), fluroquinolone-susceptible; FQ(R), fluoroquinolone-resistant; SOC standard of care

The INSPIRE TB study is a pragmatic, multicentre, randomised, controlled, open-label non-inferiority trial to evaluate the efficacy and safety of seven 9-month oral regimens compared to a 9-month standard of care (SOC) regimen in RR-TB participants susceptible to fluoroquinolones, and a bedaquiline-containing 9-month oral regimen compared to a 20-month conventional regimen in RR-TB participants resistant to fluoroquinolones (shown in Fig. 1).

### Setting

The INSPIRE TB study is a multicentre trial led by the National Medical Center for Infectious Diseases at Huashan Hospital, Fudan University. The study is being conducted across a nationwide network of over 40 clinical sites in China. Participating sites are selected with the following criteria: high burden of RR-TB; experience of treating drug-resistant TB; experience of managing adverse events during drug-resistant TB treatment; adequate supply of regimen drugs; laboratory equipped for microbiology culture and molecular diagnostics.

### Study regimens

#### In participants susceptible to fluoroquinolones

RR-TB patients susceptible to fluoroquinolones are identified with the Xpert MTB/XDR assay (Cepheid; Sunnyvale, CA, USA). Experimental arms are seven five-drug oral regimens composed of agents selected from the following: bedaquiline, linezolid, a fluoroquinolone (moxifloxacin or levofloxacin), cycloserine, clofazimine and pyrazinamide. Composition of the regimens are described in Table 1. All regimens contain pyrazinamide except for regimen A1 and regimen B-SOC. To minimize potential toxicity, each regimen includes no more than two major QT-prolonging drugs (bedaquiline, clofazimine and moxifloxacin). Treatment duration of the experimental arms is 9 months. A 2-month extension of treatment is allowed with the presence of cavities at month 9 or in case of a positive culture at month 2. Baseline molecular drug susceptibility test (DST) of pyrazinamide will be performed using the whole gene sequencing (WGS) technique. The result of pyrazinamide molecular DST will be interpreted by technical staff at central laboratory of Huashan Hospital, Fudan University according to criteria published previously [9, 10]. Once a participant is proved resistant to pyrazinamide by WGS results obtained at baseline, pyrazinamide will be discontinued with no need for extra drug replacement.

The control regimen for fluroquinolone-susceptible participants is the current SOC regimen recommended by the national guidelines [11]. The SOC regimen comprises 1–2 drugs from group A (bedaquiline, linezolid, moxifloxacin or levofloxacin) and a drug from group B (clofazimine, cycloserine) along with ethambutol, pyrazinamide and high-dose isoniazid. Composition of the study regimen and dosing is described respectively in Tables 1 and 2. Treatment duration is 9 months in total, including a 4-month intensive phase and a 5-month continuation phase. The intensive phase could be extended to 5 months or 6 months if smear conversion has not occurred by month 4 or month 5.

**Table 1:** Description of trial regimens

Group		Regimen	Duration (month)	Status
FQ(s)^a^	A-SOC	4 Bdq (Lzd) + Lfx (Mfx) + Cfz (Cs) + Pto + E + H_h_ + Z/5Lfx (Mfx) + Cfz (Cs) + E + Z	9	Recruiting
	A1	Bdq + Lfx (Mfx) + Lzd + Cs + Cfz	9	Recruiting
	A2a	Bdq + Mfx (Lfx) + Lzd + Cs + Z	9	Recruiting
	A2b	Bdq + Mfx (Lfx) + Lzd^b^ (2 months) + Cs + Z	9	Closed following protocol amendment
	A2c	Bdq + Mfx (Lfx) + Lzd^c^ + Cs + Z	9	Closed following protocol amendment
	A3	Bdq + Lfx (Mfx) + Lzd + Cfz + Z	9	Recruiting
	A4	Bdq + Lfx (Mfx) + Cfz + Cs + Z	9	Recruiting
	A5	Mfx (Lfx) + Lzd + Cfz + Cs + Z	9	Closed following protocol amendment
FQ(r)	B-SOC	6Bdq + Lzd + Cs + Cfz /14Lzd + Cs +Cfz	20	Enrolment completed
	B1	Bdq + Lzd + Cs + Cfz + Z	9	Enrolment completed

Abbreviations: Bdq, bedaquiline; Cfz, clofazimine; Cs, cycloserine; Lzd, linezolid; Mfx, moxifloxacin; Lfx, levofloxacin; Z, pyrazinamide; H_h_, high-dose isoniazid; E, ethambutol; Pto, prothionamide; FQ(s), fluroquinolones susceptible; FQ(r), fluroquinolones resistant

^a^ Drug susceptibility of fluroquinolones is determined with Xpert MTB/XDR

^b^ Treatment duration of linezolid is 2 months in regimen A2b

^c^ Treatment duration of linezolid is 9 months and dose of linezolid is halved after 2 months in regimen A2c

**Table 2:** Regimen drug doses by weight bands and usage

Drug	Weight band (kg)		Usage
	30–50 kg (mg/d)	> 50 kg (mg/d)	
Bedaquiline	400 mg daily for 2 weeks and then 200 mg three times a week		With meal
Linezolid ^a^	600 mg		Once daily, before or after meal
Moxifloxacin	400 mg		Once daily, before or after meal
Levofloxacin	500 mg	750 mg	Once daily, before or after meal
Clofazimine	100 mg		Once daily, before or after meal
Cycloserine	500 mg	750 mg	Once or divided in two doses, before or after meal
Pyrazinamide	1500 mg		Once daily, before or after meal
Prothionamide	500 mg	750 mg	Once or divided in two doses, before or after meal
Ethambutol	750 mg	1000 mg	Once daily, before or after meal
Isoniazid	400 mg	600 mg	Once daily, before or after meal

^a^ In regimen A2b, treatment dosage and duration of linezolid is 600 mg for 2 months. In regimen A2c, linezolid is routinely modified to 300 mg daily after 2 months of treatment and remains the same dose in subsequent treatment

#### In participants resistant to fluoroquinolones

Pre-XDR TB participants are identified with the Xpert MTB/XDR assay prior to treatment initiation. The experimental arm is a 9-month regimen consisting of bedaquiline, cycloserine, clofazimine, linezolid and pyrazinamide. Treatment could be extended to 11 months if cavities are present at month 9 or in case of a positive culture at month 2. Pyrazinamide will be discontinued from the study regimen if baseline WGS results reveal pyrazinamide resistance.

The comparator is a conventional longer regimen consistent with the national guidelines [11], including bedaquiline, cycloserine, clofazimine and linezolid. Treatment duration is 20 months, with a 6-month intensive phase and a 14-month continuation phase. Composition of the study regimen and dosing is described respectively in Tables 1 and 2.

#### Eligibility criteria

Participants eligible for the INSPIRE TB study must fulfil all inclusion criteria:

1. Male or female participants aged 16–75 years old, with weight over 30 kg, regardless of HIV status.
2. Pulmonary TB with rifampicin resistance diagnosed with either phenotype DST (obtained within 60 days prior to randomisation) or World Health Organization (WHO)-approved rapid molecular diagnostic testing [12].
3. Signed informed consent form (ICF).

Participants will be excluded from the trial if they meet any of the following criteria:

1. Participants with known allergies, hypersensitivity, or contraindication to any of the study drugs as described in the Supplementary material.
2. Participants with central nervous system TB, tuberculous osteomyelitis and arthritis, or hematogenous disseminated pulmonary TB.
3. Participants known to be pregnant or breastfeeding at the time of enrolment.
4. Participants who have received second-line anti-TB drugs for more than 14 days prior to randomisation, including bedaquiline, moxifloxacin, levofloxacin, linezolid, cycloserine and clofazimine.
5. Participants in critical condition and with a physician-estimated survival of less than 3 months.

#### Recruitment process

RR-TB patients diagnosed with molecular or phenotypic DST are identified by investigators and clinical staff at research sites. Investigators then explain the study regimens and follow-up schedule, as well as potential risks and benefits to participants who agree to eligibility assessment. After fully informed consent, participants are involved in the screening process, including collection of medical history and personal information, physical examination and baseline clinical evaluation. Participants who meet the eligibility criteria are included in the trial and the Xpert MTB/XDR assays are performed to determine fluoroquinolone susceptibility.

#### Randomisation

Randomisation is based on an online system (REDCap) operated remotely at Huashan Hospital, Fudan University and is stratified by research sites. Prior to randomisation, patient eligibility is re-confirmed through a comprehensive review of medical history. An individualized eligibility assessment was documented in the electronic case report form, capturing detailed data on drug susceptibility, contraindications, concomitant medications, and treatment tolerance, as specified in the Supplementary Materials. Patients with known resistance or contraindications to regimen components are excluded from the randomisation procedure and receive guideline-based standard care. Eligible participants will be stratified into fluroquinolone-susceptible arms (group A) and fluoroquinolone-resistant arms (group B) based on baseline Xpert MTB/XDR results.

In group A, the randomisation ratio between the regimen A-SOC and each experimental arm is 1:1. Enrolment into the experimental arm will be ceased once its planned sample size is reached. Participants resistant to fluoroquinolones are randomised to regimen B1 and regimen B-SOC in a 1:1 ratio.

In response to updated RR-TB treatment guidelines and emerging evidence, this trial was strategically optimized in 2024. The initial design included a bedaquiline-free arm (regimen A5). Following WHO’s endorsement of bedaquiline-based oral shorter regimens [13, 14], the study protocol was amended in version 6.0 (November 2024) to discontinue the enrolment of regimen A5, ensuring sufficient sample size for evaluation of bedaquiline-containing arms. In addition, randomisations to regimen A2b and A2c were halted based on two primary considerations. First, a recently published multicentre randomised controlled trial demonstrated the non-inferior efficacy of a nearly identical structured linezolid dose-reduction strategy [15], thereby compromising the novelty of our pre-planned analysis. Second, enrolment has progressed more slowly than we initially anticipated. Therefore, a strategic decision was made to concentrate limited resources to prioritize the remaining arms to address critical evidence gaps in RR-TB treatment.

#### Follow-up duration and participant timeline

The baseline visit includes collection of medical history, a physical examination, evaluation of symptoms and signs, laboratory tests, a 12-lead electrocardiogram, visual acuity assessment, assessment for peripheral neuropathy, smear, culture, and the Xpert MTB/XDR assay. During the treatment period, follow-up visits are scheduled monthly until treatment completion. After treatment, visits are conducted every three months in the first six months, and then every six months until 21 months after randomisation. Additional visits could be conducted as clinically needed. Follow-up assessment during treatment and post-treatment assessment are shown in Table 3. MTB strains are collected and sent to the central lab for whole genome sequencing at baseline and post-baseline visits as needed.

**Table 3:** Schedule of enrolment, interventions, and assessments

	Baseline	Treatment Period						Post-treatment Period		
		monthly						M12	M15	EOS^#^
Timepoint (month)	-2 weeks to M0	M1	M2	M3		…	EOT^**^			
Inform consent	**×**									
Eligibility assessment	**×** ^*^									
Treatment allocation	**×**									
Susceptibility testing for rifampicin	**×**									
Rapid molecular test for FQ resistance	**×**									
Treatment adherence assessment		**×**	**×**	**×**		**×**	**×**			
Evaluation of signs and symptoms	**×**	**×**	**×**	**×**		**×**	**×**	**×**		**×**
Sputum Tests (smear and culture)	**×**	**×**	**×**	**×**		**×**	**×**	**×**		**×**
Electrocardiogram	**×**	**×**	**×**	**×**		**×**	**×**	if indicated		
Routine blood tests and chemistry	**×**	**×**	**×**	**×**		**×**	**×**	if indicated		
Chest Radiography	**×**	if indicated			**×**			if indicated		
Haemoglobin A1c	**×**	Every three months in participants with diabetes								
TSH, FT3, FT4	**×**	monthly if on prothionamide								
HIV test, hepatitis B and C	**×**									
Pregnancy test	**×**									
Visual acuity assessment	**×**	if indicated						if indicated		
Assessment for peripheral neuropathy	**×**	if indicated						if indicated		
SDS and SAS	**×**	if indicated						if indicated		

Abbreviation: EOT: end of treatment; EOS: end of study; TSH: thyroid-stimulating hormone; FT3: free triiodothyronine; FT4: free thyroxine; SDS: self-rating depression scale; SAS: self-rating anxiety scale

^*^ Sputum rapid molecular test for rifampicin resistance should be performed during screening for eligibility assessment, and rapid molecular test for fluoroquinolone resistance should be acquired before randomisation for stratification

^**^ Timepoint of EOT in Regimen A1, A2, A3, A4, A5 and B1 is 9 months. Timepoint of EOT in Regimen B-SOC is 20 months

^#^ Timepoint of EOS is 21 months after randomisation

#### Treatment compliance

At each visit, study medications are prescribed and dispensed to participants by a research nurse or doctor to ensure correct treatment. Each participant is provided with a medication diary for self-documentation. During follow-up visits, comprehensive adherence assessments are conducted by research doctors, verifying drug types, dosages, and any missed or incorrect doses, with all data recorded in the case report form. Additionally, the central research team conducts regular cross-checks with site investigators to verify the total medication consumption for each participant to address any instances of missed or reduced doses.

#### Sample size assumptions

The sample size calculation was performed in PASS 11 system (NCSS, Version 11.0.10). In an individual patient data meta-analysis of 13,273 MDR/RR-TB patients from 38 countries, the treatment success rate of the bedaquiline-containing oral shorter regimen is 73% [16]. We thus assume a 70% favourable rate in the control arm and 80% in the experimental arms in fluoroquinolone susceptible patients. Since multiple experimental groups are simultaneously compared with the control group, the family-wise type I error will be controlled by a fixed sequence approach. The experimental groups are tested sequentially in a pre-specified order, and if any group fails to demonstrate non-inferiority, testing for subsequent groups will be discontinued. All the previous comparisons will be done at the one-sided alpha level of 2.5%. Therefore, a sample size of 73 per arm will afford 80% power to establish non-inferiority of seven experimental regimens with a non-inferiority margin of 10% at a one-sided α level of 2.5%. The number excluded from the modified intention-to-treat population is estimated to be 30% and therefore the recruitment target is increased to 104 per arm. The total sample size of fluoroquinolone-susceptible RR-TB participants is 832.

By November 2024, randomisation to regimen A2b, A2c as well as A5 has been ceased following protocol amendment. The actual number of enrolled participants at the time of early closure were 73, 72, 92 respectively in regimen A2b, A2c and A5. Therefore, the total sample size of fluoroquinolone-susceptible RR-TB participants have been adjusted to 520 (including regimen A1, A2a, A3, A4 and A-SOC).

In fluoroquinolone-resistant RR-TB participants, we assume that 75% of participants in the experimental arm (regimen B1) and 65% of participants in the control arm (regimen B-SOC) will achieve a favourable outcome. A sample size of 82 per arm will afford 80% power to establish non-inferiority with a margin of 10% and a one-sided α level of 0.025. The number excluded from the modified intention-to-treat population is estimated to be 30% and therefore the recruitment target is increased to 117 per arm. The total sample size of fluoroquinolone-resistant participants is 234.

As of September 2025, enrolment for the fluoroquinolone-resistant group has completed, while recruitment of fluoroquinolone-susceptible participants remains ongoing.

#### Outcome measurements

The primary efficacy outcome is the proportion of participants with a favourable outcome at 21 months after randomisation. The primary analysis will be conducted in the modified intention-to-treat (mITT) population in fluoroquinolone-susceptible and fluoroquinolone-resistant arms respectively. Definition of treatment outcomes is summarized in Table 4.

**Table 4:** Definitions of primary outcome

Outcome definitions
**Favourable outcome**: The outcome will be classified as favourable if not previously classified as unfavourable, and one of the following is true: (1) The last two culture results are negative. These two cultures must be taken from respiratory samples collected on separate visits at least 7 days apart. The latest culture sample collected between month 21 and 23. (2) The last culture result (from a respiratory sample collected between month 21 and 23) is negative; and there is no other post-baseline culture result; and bacteriological, radiological and clinical evaluation is favourable. (3) There is no culture result collected between month 21 and 23 or the result of that culture is positive due to laboratory cross contamination; and the most recent culture result is negative; and bacteriological, radiological and clinical evaluation is favourable.
**Unfavourable outcome**: (1) At least one of the last two cultures (taken from respiratory samples collected on separate visits at least 7 days apart) is positive and laboratory cross contamination is ruled out. The latest between month 21 and month 23. (2) There is no culture result collected between month 21 and 23 or the result is positive due to laboratory cross contamination; and - bacteriological, radiological and clinical evaluation is unfavourable. OR - the most recent culture result is positive in the absence of laboratory cross contamination. (3) There is no culture result collected between month 21 and 23 or the result is positive due to laboratory cross contamination; and - either there is no other post-baseline culture result, or the penultimate culture result is positive due to laboratory cross contamination. OR - bacteriological, radiological and clinical evaluation is not available. (4) The last culture result (from a respiratory sample collected between month 21 and 23) is negative; and either there is no other post-baseline culture result, or the penultimate culture result is positive due to laboratory cross contamination; and bacteriological, radiological and clinical evaluation is unfavourable. (5) The last culture result (from a respiratory sample collected between month 21 and 23) is negative; and either there is no other post-baseline culture result, or the penultimate culture result is positive due to laboratory cross contamination; and bacteriological, radiological and clinical evaluation is not available. (6) Participant whose treatment regimen needed to be terminated or permanently changed ^a^ to a new regimen. (7) Treatment extension that does not meet protocol-defined extension criteria. (8) Initiation of a new RR-TB treatment regimen after the end of the allocated study regimen and before month 21 (relapse). (9) Loss to follow-up ^b^. (10) Death from any cause.

^a^ A permanent regimen change is constituted by any of the following: [1] adding or replacing two or more drugs in the allocated regimen; [2] adding second-line injectables in the allocated regimen; [3] replacing a group C drug with a group B drug (clofazimine, cycloserine) not originally included; [4] replacing a group B drug with a group A drug (moxifloxacin, levofloxacin, linezolid, bedaquiline) not originally included

^b^ Loss to follow-up is defined in case that a participant fails to complete two or more consecutive scheduled visits and cannot be reached after at least three phone calls and one home interview

Main secondary outcomes are measured in fluoroquinolone-susceptible and fluoroquinolone-resistant arms respectively. Secondary outcomes are defined as follows:

1. Time to sputum smear conversion in the mITT population.
2. Time to sputum culture conversion in the mITT population.
3. The proportion of participants with a favourable outcome at 21 months post-randomisation in the per-protocol (PP) population.
4. The proportion of participants with grade 3 or higher adverse event (AEs), serious adverse events (SAEs) at 21 months post-randomisation in the safety population.
5. All-cause mortality and treatment relevant mortality at 21 months post-randomisation in the safety population.
6. The proportion of participants with treatment relevant SAEs at 21 months post-randomisation in the safety population.
7. The proportion of participants with grade 3 or higher QTc prolongation in the safety population.
8. The proportion of participants experiencing permanent drug discontinuation or replacement due to QTc prolongation in the safety population.

Exploratory outcome measures are defined as the following:

1. The proportion of participants with a favourable outcome at 21 months after randomisation in regimen A2a, A2b and A2c in the mITT population.
2. The proportion of participants with grade 3 or higher myelosuppression, peripheral neuropathy and optic neuropathy in regimen A2a, A2b and A2c in the safety population.
3. The proportion of participants with linezolid discontinuation or dose reduction due to AE in regimen A2a, A2b and A2c in the safety population.

#### Statistical considerations and analysis population

Analysis populations included the intention-to-treat (ITT) population, the modified intention-to-treat population and the per-protocol population. The ITT population includes all participants undergoing randomisation. The mITT population is defined as participants who undergo randomisation and had a culture positive for *Mycobacterium tuberculosis* collected at baseline or within two weeks of randomisation, excluding those with baseline isolates susceptible to rifampicin based on phenotypic DST. Additionally, for the mITT analysis, participants in Group A with fluoroquinolone-resistant baseline isolates confirmed with phenotypic DST will be excluded. Conversely, those in Group B with fluoroquinolone-susceptible isolates will be excluded. Participants who undergo randomisation in error or do not receive any study treatment after randomisation are also excluded from the mITT population. The PP population is derived from the mITT population, excluding those with inadequate treatment, defined as having completed less than 80% of the prescribed doses within 120% of the planned duration. Participants who discontinue treatment due to treatment failure, adverse events, or death will not be excluded from the PP population. Safety analyses will be conducted in the safety population, which includes all participants who receive at least one dose of the study regimen, and will be analysed according to the treatment actually administered.

The primary efficacy analysis will be conducted in the mITT population, and efficacy outcomes will be reported as supportive analyses from both the PP and ITT populations. The absolute between-group difference with 95% confidence interval (CI) in the proportion of participants with a favourable outcome will be calculated. The non-inferiority of an experimental arm versus the control arm will be established if the upper bound of the 95% CI is less than 10% (the margin of non-inferiority). In group A, multiple experimental arms are compared to the control regimen. Type I error rate will be controlled with a pre-planned fixed sequence method. First, the regimen with the highest expected treatment success rate will be compared to the control. If non-inferiority is established, comparisons will proceed sequentially to the next experimental regimen in descending order of expected efficacy. For any regimen that demonstrates non-inferiority, a subsequent test for superiority over the control will be performed.

Patient characteristics will be described with median and range (or interquartile range), or numbers and percentages. The median time to smear and culture conversion will be estimated in each arm using the Kaplan-Meier method and between-group difference will be compared with the log-rank test. Proportion of participants with any grade 3–5 adverse event, any serious adverse event (SAE) and any treatment-related SAE will be described with numbers and percentages.

#### Safety

All preferred terms of adverse events (AEs), system organ class and grading of severity are in accordance with the Common Terminology Criteria for Adverse Events (CTCAE) version 5.0. Adverse events will be collected from the signing of the ICF until the participants’ last known visit in the trial. All AEs will be recorded in the electrical case report form. Reporting framework are as the followings: terms of an AE, severity grading for each AE, its duration, relationship to the study regimen, whether the study regimen is maintained or discontinued after an AE, any dose adjustment of regimen drugs resulted from an AE, any concomitant treatment related to AE management, and whether it is a serious adverse event. All death cases during the study are reported and probable causes of death will be reviewed by an independent review committee and categorized as related to anti-tuberculosis treatment, related to tuberculosis, and other/unknown causes. An independent Data and Safety Monitoring Board (DSMB) has been established to periodically review efficacy and safety data. Based on these reviews, the DSMB will provide recommendations regarding trial continuation, protocol amendments, or early termination of study arms.

#### Data collection, monitoring and management

Clinical staff at research sites will record data into a case report form designed for the study. Data are then entered into a web-based system. Regular check-ups for data accuracy and consistency will be performed by designated research team members for quality control purpose. Any modification to the data will leave editing traces in the online database.

Central data management team will regularly review data for completeness and accuracy. Queries with respect to missing values and inconsistent data are sent to participating research sites via the web-based database system. Data correction or clarification is requested. After the final examination for data accuracy and completeness, database will be locked down for generation of data extracts and final analysis.

#### Confidentiality

Participants will be identified with a subject number in all study documents, data collection forms and all laboratory specimens. Paper documents will be stored and locked in the trial office. Digital data are protected through password-protected electronic database. Access will be granted to authorized research investigators and staff only.

#### Patient and public involvement

This multicentre, open-label, randomised controlled trial has implemented treatment regimens and study procedures that strictly follow current WHO guidelines, expert consensus and standardised clinical trial protocols for drug resistant tuberculosis. Therefore, patient and public members were not directly involved in the design and conduct of the study. However, patient and public partners will be engaged in the dissemination of trial results. Patient representatives will be supported in sharing their treatment experiences and participating in media engagements. All findings will be communicated to study participants and public members in an accessible format through domestic media platforms.

## Discussion

INSPIRE TB is a pragmatic, multicentre, randomised, non-inferiority open-label trial aiming to identify effective 9-month oral regimens in fluoroquinolone-susceptible and fluoroquinolone-resistant RR-TB patients respectively. A pragmatic design is chosen to assure the evaluation of the candidate regimens in a diverse population, regardless of HIV status in over 30 clinics and hospitals around China under routine care. As current MDR/RR-TB treatment guidelines have endorsed several bedaquiline-based oral regimens, the INSPIRE TB trial aims to address a critical gap in the implementation of bedaquiline-containing regimens in China and generate robust evidence for national RR-TB treatment guidelines.

The year 2024 has witnessed major updates in MDR/RR-TB treatment. Based on the results of two clinical trials, WHO has announced recently that the 6-month BDLLC (bedaquiline, delamanid, levofloxacin, linezolid and clofazimine) in the Beat-TB study and the 9-month of modified oral regimens in the endTB trial may be used pragmatically instead of longer regimen [14, 17]. In the endTB trial, four efficacious 9-month oral regimens (BLMZ, BLLfxCZ and BDLLfxZ) were compared to the local standard of care regimens and favourable outcomes were attained in over 80% of participants, which expanded treatment options for RR-TB [8]. The TB PRACTECAL study also adopted a multi-arm and multi-stage design to assess various candidate regimens including bedaquiline, pretomanid and linezolid [18, 19]. Despite the accumulation of global evidence, there remains a significant gap in the evaluation for bedaquiline-containing oral shorter regimens under trial settings in China. Our study is expected to fill this gap. In addition, the INSPIRE TB study employs a multi-arm design, enabling the evaluation of four potential 9-month oral regimens (following the cessation of randomisation to regimen A2b, A2c and A5) in participants with fluoroquinolone-susceptible RR-TB. This approach would provide critical insights into the implementation of oral regimens with various drug combinations domestically.

Similar to the endTB trial, this study adopts a pragmatic approach to evaluate candidate regimens in real world clinical settings. First, the INSPIRE TB study applies minimal inclusion and exclusion criteria, enrolling a broad spectrum of patients with varying disease severity, comorbidities and HIV status. Second, this trial is conducted across multiple clinics and hospitals nationwide by staff engaged in routine clinical care, thereby simulating real-world conditions. Third, the study protocol allows appropriate drug modifications in response to safety events or treatment failure, aligning with standard RR-TB management. As the WHO has called for evaluation of newly-recommended regimens across different regions and populations [13, 20], our study aims to contribute critical evidence to address this gap.

The INSPIRE TB trial initially incorporated regimen A2a, A2b and A2c to investigate a structured linezolid dose-reduction strategy in RR-TB treatment. Currently, there remains no consensus on the optimal dosing and treatment duration of linezolid, with strategies varying widely across trials. The ZeNix trial previously evaluated four linezolid doses (1200 mg daily for 26 weeks, 1200 mg daily for 9 weeks, 600 mg daily for 26 weeks, and 600 mg daily for 9 weeks), with safety and efficacy outcomes favouring the 600 mg/day 26-week regimen [21]. In the TB‑PRACTECAL trial, linezolid was administered at 600 mg per day for 16 weeks, followed by 300 mg per day for 8 weeks [22]. Our initial design planned to assess three linezolid dosing group: 600 mg daily for 9 months, 600 mg daily for 2 months, 600 mg daily for 2 months followed by 300 mg daily. However, during the trial enrolment phase, emerging evidence from clinical trials adopting similar strategy has prompted a re-evaluation of study design [15]. Concurrently, trial recruitment has been slower than we anticipated, which necessitated the discontinuation of randomisation to regimens A2b and A2c to ensure adequate sample size for the remaining arms. Despite this, safety and efficacy data from regimen A2b, A2c will be reported as exploratory analyses to provide knowledge for optimal linezolid dosing within a 9-month bedaquiline-based oral regimen for fluoroquinolone-susceptible RR-TB.

The current study has several limitations. First, the exclusion of children, adolescents, and pregnant women limits the generalizability of the findings to these populations. Recently WHO has endorsed the use of bedaquiline in oral shorter regimens in children and adolescents with drug-resistant tuberculosis in all age groups [23]. And second-line treatment in pregnant women with RR-TB has been proven safe in cohort study [24, 25]. However, evidence on the implementation of new and repurposed drugs in such populations remains limited and for safety concern they are excluded from the study. Secondly, the open‑label design introduces the possibility of assessment bias, as outcomes are determined at the investigators’ discretion. To mitigate potential bias, standardised outcome evaluation rules are applied across all arms, and outcomes must be verified with bacteriological, radiological and clinical evidence documented in the database. Third, the trial does not include a BPaL-based regimen as either a control or experimental arm, as pretomanid is not domestically available, which may affect the generalizability of the findings to settings where BPaL regimen is used. Therefore, future studies to evaluate pretomanid-containing oral regimens for RR-TB in China will be essential.

Overall, progress and advancements in MDR/RR-TB treatment policy and guidelines has been limited. Although recent evidence from randomised controlled trials has prompted updates in treatment guidelines, there remains an urgent need to re-validate the efficacy and safety of those regimens in a larger, more diverse population within pragmatic real-world settings. Hopefully the INSPIRE TB study will improve treatment options and provide high-quality evidence of the efficacy and safety of four 9-month oral regimens in fluoroquinolone-susceptible RR-TB patients and one 9-month bedaquiline-containing oral regimen in Pre-XDR TB patients.

## Supplementary Information

Below is the link to the electronic supplementary material.

Supplementary Material 1

Supplementary Material 2

## Data Availability

No datasets were generated or analysed during the current study.

## Abbreviations

AEs: Adverse events
CI: Confidence interval
CRF: Case report form
DST: Drug susceptibility testing
CTCAE: Common terminology criteria for adverse events
FQ(S): Fluoroquinolone-susceptible
FQ(R): Fluoroquinolone-resistant
HIV: Human immunodeficiency virus
ITT: Intention-to-treat
MDR-TB: Multidrug-resistant tuberculosis
mITT: Modified intention-to-treat
MTB: Mycobacterium tuberculosis
NMCID: National Medical Centre for Infectious Diseases
PP: Per-protocol
Pre-XDR TB: Pre-extensively drug-resistant tuberculosis
QTc: QT interval corrected for heart rate
RCT: Randomised controlled Trial
RR-TB: Rifampicin-resistant tuberculosis
SAEs: Serious adverse events
SOC: Standard of care
WGS: Whole-genome sequencing
WHO: World health organization
