# Supplementary material for: Innovating shorter, all-oral, precise, individualized treatment regimen for rifampicin-resistant tuberculosis (INSPIRE TB): study protocol for a pragmatic randomised controlled trial
Source: BMC Infect Dis. 2026 Mar 17;26:828. doi: 10.1186/s12879-026-13017-y (PMC13107636; doi:10.1186/s12879-026-13017-y)
Supplement: Supplementary file 2 — Supplementary Material 2 [file 12879_2026_13017_MOESM2_ESM.docx]

Table S1 Instructions for drug assessment

| Drug | | Contraindications | Other risks identified by physicians | Other considerations^*^ |
| --- | --- | --- | --- | --- |
| Bdq | | □ History of allergy/hypersensitivity; □ Baseline QTc >500ms (confirmed by repeat ECG); □ Clinically significant ventricular arrhythmia. | □ Patients at increased risk of QT interval prolongation (history of congenital long QT syndrome; history of hypothyroidism and bradyarrhythmia; history of decompensated heart failure; baseline hypokalaemia; hypocalcaemia; hypomagnesemia); □ History of severe hepatic insufficiency or baseline liver enzyme elevation >5 times; □ History of renal insufficiency (GFR <30 mL/min) or dialysis patients; □ Patients taking the antiretroviral drugs (e.g. efavirenz, ritonavir); □ Patients taking the antiviral drug: ledipasvir/sofosbuvir; □ Patients taking strong CYP3A inducers (apalutamide, enzalutamide, carbamazepine, phenytoin sodium, rifamycin) or strong CYP3A4 inhibitors (voriconazole, itraconazole, ketoconazole, posaconazole, telithromycin, clarithromycin). | □ |
| FQs | **Lfx** | □ History of allergy/hypersensitivity. | □ History of psychiatric or neurological disorders (anxiety, depression, epilepsy, convulsions); □ Need for dose adjustment in renal insufficiency; □ Patients at increased risk for prolongation of the QT interval (history of congenital long QT syndrome; history of hypothyroidism and bradyarrhythmia; history of decompensated heart failure; baseline hypokalaemia, hypocalcaemia, hypomagnesemia); □ History of myasthenia gravis; □ History of fluoroquinolone-related tendon disease. | □ |
|  | **Mfx** | □ History of allergy/hypersensitivity. | □ Patients with psychiatric or neurological disorders (anxiety, depression, epilepsy, convulsions); □ History of severe hepatic insufficiency or baseline hepatic enzyme elevation > 5 times; □ Patients at increased risk of QT interval prolongation (history of congenital long QT syndrome; history of hypothyroidism and bradyarrhythmia; history of decompensated heart failure; baseline hypokalaemia, hypocalcaemia, hypomagnesemia); □ History of severe myasthenia gravis; □ History of fluoroquinolone-related tendon disease. | □ |
| Lzd | | □ History of allergy/hypersensitivity; □ Taking or having used monoamine oxidase inhibitors (MAOIs) within two weeks of screening. | □ Visual impairment; □ Leukopenia; □ Thrombocytopenia; □ Moderate-to-severe anaemia; □ Post-chemotherapy for tumours; □ History of psychiatric or neurological disorders (anxiety, depression, epilepsy, seizures); □ History of peripheral neuropathy; □ Uncontrolled hypertension; □ Pheochromocytoma, carcinoid syndrome, or untreated hyperthyroidism; □ Taking or having used a tricyclic antidepressant, selective serotonin reuptake inhibitor, serotonin and noradrenaline reuptake inhibitor, or other monoamine oxidase inhibitors, opioid analgesics (e.g., clomipramine, amitriptyline, disopyramide, promethazine, doxepin, selegiline, trazodone, buspirone, bupropion, mirtazapine, methadone, fentanyl pethidine hydrochloride) within two weeks of screening. | □ |
| Cs | | □ History of allergy/hypersensitivity; □ History of psychiatric disorders such as severe anxiety, depression or history of epilepsy or convulsive seizures. | □ Dose adjustment required for renal insufficiency; □ History of severe hepatic insufficiency or baseline hepatic enzyme elevation > 5 times; □ History of alcohol abuse. | □ |
| Cfz | | □ History of allergy/hypersensitivity. | □ History of severe hepatic insufficiency or baseline hepatic enzyme elevation > 5 times; □ History of gastrointestinal disorders; □ Patients at increased risk for prolongation of the QT interval (history of congenital long QT syndrome; history of hypothyroidism and bradyarrhythmia; history of decompensated heart failure; baseline hypokalaemia, hypocalcaemia, hypomagnesemia). | □ |
| Pto | | □ History of allergy/hypersensitivity; □ Pregnancy. | □ History of psychiatric or neurologic disorders (anxiety, depression, epilepsy, convulsions); □ History of chronic liver disease or baseline hepatic enzyme elevation > 5 times. | □ |
| Z | | □ History of allergy/hypersensitivity; □ History of severe hepatic impairment. | □ Baseline liver enzyme elevation > 5 times; □ Renal insufficiency requiring dose adjustment; □ History of gout or active gout. | □ |
| E | | □ History of allergy/hypersensitivity; □ History of severe hepatic impairment. | □ With gout; □ History of renal insufficiency (GFR <30 mL/min) or patients on dialysis; □ With optic neuritis. | □ |
| H_h_ | | □ History of allergy/hypersensitivity; □ History of severe hepatic impairment；  □ Detection of both *inhA* promoter and *katG* mutations. | □ History of psychiatric or neurological disorders (anxiety, depression, epilepsy, convulsions); □ Baseline hepatic enzyme elevation > 5 times; □ History of alcohol abuse; □ History of peripheral neuropathy. | □ |

Abbreviations: Bdq, bedaquiline; Cfz, clofazimine; Cs, cycloserine; Lzd, linezolid; Mfx, moxifloxacin; Lfx, levofloxacin; Z, pyrazinamide; H_h_, high-dose isoniazid; E, ethambutol; Pto, prothionamide; FQs, fluroquinolones

^*^Other considerations include but are not limited to drug resistance (identified by Xpert MTB/XDR), treatment acceptability (i.e., drug preference, treatment fee etc.), tolerability (i.e., intolerance to certain side effects, refusing to use for certain side effects etc.)
